# Teaching-learning strategies on patient safety in higher education institutions: a scoping review

**DOI:** 10.1590/0034-7167-2024-0270

**Published:** 2025-03-10

**Authors:** Nicole Ferreira, Vanessa Albuquerque Alvim de Paula, Marcelo Carneiro, Vilanice Alves de Araújo Püschel, Fábio da Costa Carbogim, André Luiz Silva Alvim

**Affiliations:** I Universidade Federal de Juiz de Fora. Juiz de Fora, Minas Gerais, Brazil; II Universidade de Santa Cruz do Sul. Santa Cruz do Sul, Rio Grande do Sul, Brazil; III Universidade de São Paulo. São Paulo, São Paulo, Brazil; IV Centro Brazileiro para o Cuidado à Saúde em Evidências: Centro de Excelência do JBI. São Paulo, São Paulo, Brazil

**Keywords:** Patient Safety, Universities, Education, Learning, Review, Seguridad del Paciente, Universidades, Educación, Aprendizaje, Revisión

## Abstract

**Objectives:**

to map the scientific production on teaching-learning strategies related to patient safety in higher education institutions across Nursing, Pharmacy, Medicine, and Dentistry programs.

**Methods:**

this scoping review follows the Joanna Briggs Institute (JBI) guidelines and the PRISMA Extension for Scoping Reviews recommendations. The selection of studies was performed using databases, grey literature, and reverse searching, conducted by two independent and blinded reviewers.

**Results:**

nineteen studies were included, the majority of which were conducted at the undergraduate level, primarily among nursing students. The identified teaching-learning strategies included clinical simulation, courses, competency assessment, and expository methods. Additional strategies employed were workshops, structured objective clinical examinations, flipped classrooms, team testing, questionnaire applications, dramatization, and games.

**Conclusions:**

the most commonly used teaching-learning strategies were clinical simulation and courses on patient safety.

## RESUMEN

**Objetivos:**

mapear la producción científica sobre estrategias de enseñanza-aprendizaje relacionadas con la seguridad del paciente en instituciones de educación superior en los cursos de Enfermería, Farmacia, Medicina y Odontología.

**Métodos:**

se trata de una revisión de alcance siguiendo las directrices del *Joanna Briggs Institute* (JBI) y las recomendaciones de PRISMA *Extension for Scoping Reviews*. La selección de los estudios se realizó en bases de datos, literatura gris y búsqueda inversa, llevada a cabo por dos revisores de forma independiente y a ciegas.

**Resultados:**

se incluyeron 19 estudios, la mayoría realizados en el ámbito de la educación de pregrado, principalmente entre estudiantes de Enfermería. Las estrategias de enseñanza-aprendizaje identificadas fueron: simulación clínica, cursos, evaluación por competencias y método expositivo. Además de estas, se utilizaron talleres, examen clínico objetivo estructurado, aula invertida, prueba en equipo, aplicación de cuestionarios de pruebas, dramatización y juegos.

**Conclusiones:**

las estrategias de enseñanza-aprendizaje más utilizadas fueron la simulación clínica y los cursos sobre seguridad del paciente.

## INTRODUCTION

Patient safety involves the quality and efficiency of care provided to health service users^(1)^. It refers to reducing unnecessary harm to an acceptable minimum, largely dependent on scientific knowledge and the theoretical-practical skills of healthcare professionals. This includes implementing rigorous protocols, promoting a safety culture, and providing health education to reduce incidents and adverse events^(2)^.

Therefore, it is essential that higher education institutions (HEIs) incorporate strategies focused on patient safety training into their curriculum planning. Training students throughout their educational process, whether in undergraduate or graduate studies, aims to identify and prevent damages that may risk the lives and health of patients, such as medication errors, healthcare-related infections, pressure injuries, and falls^(3)^.

A study reinforces that promoting patient safety involves a multifaceted approach, which influences the learning process during training and ultimately ensures the quality of care in health services. When health professionals are encouraged by faculty members during their academic journey, they are more likely to provide safe, harm-free care^(4)^.

The teaching-learning strategies on patient safety developed by HEIs should be interdisciplinary, covering all areas of health. These strategies are conceived as procedures and behaviors chosen to facilitate the acquisition, storage, and application of knowledge. They promote the integration of theory and practice, encouraging critical reflection and continuous training of students and health professionals^(5)^.

Undergraduate courses in health fields such as Nursing, Medicine, Pharmacy, Physiotherapy, Dentistry, and other professions operating at different levels of healthcare should include specific disciplines on patient safety in their curricula. In graduate studies, it is important that the offerings of training and updates delve into clinical practice issues to enhance the skills and competencies of professionals^(4,6)^.

Researchers warn that teaching patient safety in undergraduate and graduate programs still needs improvement^(7,8)^. Content taught using various methodological approaches can be perceived by students as a priority that directly impacts health. However, the literature does not present in a single study the teachinglearning strategies on patient safety developed and employed by higher education courses. These studies are usually conducted in a unicentric manner, with small samples, and present results that cannot be generalized. This constitutes the gap that justifies this study^(3,7,8)^.

This study may foster reflections on the impact of education and its teaching-learning methodologies on the training process regarding patient safety in HEIs, especially in the fields of Nursing, Pharmacy, Medicine, and Dentistry, which have the highest number of students in the health area. Therefore, the goal is to identify learning strategies aimed at the health training process, including training sessions, workshops, and other capacity-building activities carried out in the classroom, aiming to address the challenges related to the theme in different scenarios of action.

## OBJECTIVES

To map the scientific production on teaching-learning strategies related to patient safety in HEIs across the fields of Nursing, Pharmacy, Medicine, and Dentistry.

## METHODS

### Ethical Considerations

As this study only included publications available in the literature, it was not necessary to seek approval from the Research Ethics Committee (REC).

### Study Type

This is a scoping review structured according to the JBI methodology^(9)^ and the PRISMA Extension for Scoping Reviews (PRISMA-ScR) recommendations^(10)^ to guide the conduct and writing of the study.

### Methodological Procedure

The objective, research question, inclusion criteria, and methods were predefined and aligned in a scoping review protocol, which was registered and published on the Open Science Framework (OSF) under the DOI: 10.17605/OSF.IO/YRWXN. To formulate the research question, the PCC mnemonic was adopted, where P = participants, C = concept, and C = context. For this review, the following were adopted: P = health-related courses; C = teaching-learning strategies on patient safety; and C = higher education institutions, for both undergraduate and postgraduate courses. Thus, the guiding question was defined as: What are the teaching-learning strategies on patient safety developed and implemented in health courses by HEIs?

Inclusion criteria considered publications covering analytical and descriptive studies, as well as experimental or quasi-experimental and review studies. The types of documents included were scientific articles, monographs, dissertations, and theses. In addition to database searches, researchers conducted a reverse search on all references from these studies to identify additional studies. No temporal or language restrictions were imposed. Excluded were abstracts, letters to the editor, opinion articles, studies that did not address the research question, and duplicate records in data sources.

### Data Collection and Organization

The database search was conducted on July 12, 2023, and updated on January 2, 2024, following a search strategy outlined with the help of a librarian to locate published and unpublished studies. Keywords, descriptors, and/or free terms of interest were used for the retrieval of scientific information, considering the specifics of each database and the online library. In this case, both paid and free-access articles were included. The selection of publications in grey literature was performed on Google Scholar (first 10 pages) and Open Access Theses and Dissertations (OATD) (Chart 1).

**Chart 1:** Description of search strategies for the retrieval of scientific information, Juiz de Fora, Minas Gerais, Brazil, 2024

Source	Search strategies
PUBMED	Search: (medicine/educ*) OR (“Physical Therapists/education”[Mesh]) OR (“Education, Pharmacy”[Mesh]) OR (“Education, Dental”[Mesh]) OR (“Education, Nursing”[Mesh]) AND (course) OR (discipline) AND (patient safety/educ*)
Scopus	(TITLE-ABS-KEY (“Patient Safety” AND “Teaching” OR “Universities” not “Health professionals”) AND (course) OR (discipline) AND (patient safety/educ*)
LILACS*	(“Patient Safety”) AND (“Teaching”) OR (“Educations”) AND (“Universities”) AND (db:(“LILACS”)
BDENF (*Base de Dados em Enfermagem*)	(“Patient Safety”) AND (“Teaching”) OR (“Educations”) AND (“Universities”) AND (db:(“BDENF”)
IBECS (*Índice Bibliográfico Espanhol de Ciências da Saúde*)	“Patient Safety” AND “Teaching” OR “Educations” AND “Universities” AND (db:(“IBECS”)
Index Medicus do Pacífico Ocidental (WPRO)	tw:(“Patient Safety” AND “Teaching” OR “Universities” OR “Graduation” OR “Postgraduate” not “Hospitals”) AND (db:(“WPRIM”)
Cochrane Library	“Patient Safety” AND “Teaching” OR “Educations” AND “Universities” NOT “Health professionals” NOT “Hospitals”
SciELO (Scientific Electronic Library Online)	“Patient Safety” AND “Teaching” OR “Universities” OR “Discipline” NOT “Health professionals” NOT “Hospitals”
Google Scholar	Patient Safety” AND “Teaching” OR “Universities” NOT “Health professionals”
OATD	Patient Safety AND Teaching OR Universities NOT Health professionals

* *The search strategy was conducted using descriptors in three languages (English, Portuguese, and Spanish), as LILACS is a trilingual database.*

Subsequently, a reverse search was conducted on all references and citations from the selected studies for data extraction. After executing the search strategy, the information gathered from various sources was imported into Mendeley® and duplicates were removed. Shortly thereafter, the records were imported into Rayyan – Intelligent Systematic Review software, which was used for the selection and exploration of the studies^(11)^. This stage involved reviewing the titles and abstracts according to the PCC strategy defined in this scoping review. Later, a detailed reading of the selected texts was performed by the researchers, who recorded reasons for excluding publications when necessary.

### Data Analysis

The selection process was conducted by two independent reviewers, and any discrepancies were resolved by a third researcher^(9)^. The JBI recommendations were followed for data extraction^(12)^. The information from the selected studies included in the scoping review was recorded in a specific form, which had been previously tested on the first five publications identified during the search strategy. No needs for adjustments or additions of information were identified. The variables considered were: author, year of publication, title, country of the study, language, type of study, setting, participants, sample size, learning strategy used, and main highlights.

The results were presented through tables and the PRISMA flowchart, which addressed the research question and the objectives established in this study. Data analysis was conducted using descriptive statistics, presenting absolute and relative values concerning the included publications. To facilitate the identification of studies to be discussed, the letter “E” was used as an identifier for the studies.

## RESULTS

**Figure 1:**
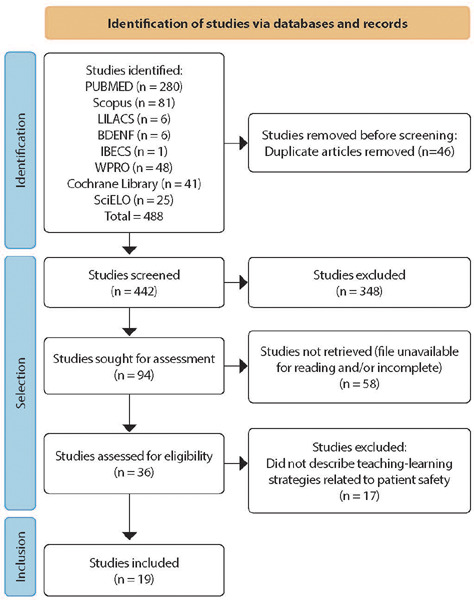
PRISMA of the articles identified and included in the scoping review, Juiz de Fora, Minas Gerais, Brazil, 2024

Figure 1 displays the PRISMA flowchart of the publications from this scoping review. A total of 488 studies were identified in the selected databases and electronic library for the study. Of these, 19 were included after screening the publications and meeting the eligibility criteria. Regarding grey literature and reverse search, the studies found were already contained in one or more of the databases included in this study and were not characterized as new research.

Chart 2 shows that the majority of the studies were published in the years 2019 (31.6%) and 2020 (21.1%). English was the predominant language of publication (94.7%), with the United States leading in research related to the topic. Various methodological approaches were employed, with variations in sample size, primarily among undergraduate students, most of whom were nursing students.

**Chart 2:** Synthesis of the studies included in the scoping review, Juiz de Fora, Minas Gerais, Brazil, 2024 (N=19)

ID*	Author/Year	Title	Country of Study	Language	Study type	Environment	Participants	Sample Size
E1	Robertson et al. (2010)^(13)^	The Use of Simulation and a Modified TeamSTEPPS Curriculum for Medical and Nursing Student Team Training	United States	English	Descriptive study	Undergraduate	Nursing and medicine students	213
E2	Lee et al. (2023)^(14)^	Evaluating a patient safety course for undergraduate nursing students: A quasiexperimental study	Canada	English	Quasiexperimental study	Undergraduate	Nursing students	107
E3	Yilmaz et al. (2020)^(15)^	The effectiveness of scenario-based learning to develop patient safety behavior in first year nursing students	Canada	English	Quasiexperimental study	Undergraduate	Nursing students	351
E4	Lee et al. (2020)^(16)^	Patient safety education in pre-registration nursing programmes in South Korea	South Korea	English	Descriptive study	Undergraduate	Nursing faculty	80
E5	Sanko et al. (2020)^(17)^	Participation in a System-Thinking Simulation Experience Changes Adverse Event Reporting	United States	English	Interventioncontrol study	Undergraduate	Nursing and medicine students	231
E6	Moloney et al. (2020)^(18)^	Fourth year nursing students’ perceptions of their educational preparation in medication management: An interpretative phenomenological study	Ireland	English	Qualitative study	Undergraduate	Nursing students	14
E7	Packard et al. (2019)^(19)^	A Synchronous Interprofessional Patient Safety Simulation Integrating Distance Health Professions Students	United States	English	Descriptive study	Undergraduate	Students from various programs	201
E8	Danko (2019)^(20)^	The Effect of an Undergraduate Perioperative Nursing Course on Safety Knowledge	United States	English	Descriptive study	Undergraduate	Nursing students	44
E9	Mbuthia et al. (2019)^(21)^	Preregistration nursing students’ perceived confidence in learning about patient safety in selected Kenyan universities	Africa	English	Descriptive cross-sectional study	Undergraduate	Nursing students	194
E10	Bajis et al. (2019)^(22)^	Pharmacy students’ medication history taking competency: Simulation and feedback learning intervention	Australia	English	Cohort study	Undergraduate	Pharmacy students	144
E11	Sakamoto et al. (2019)^(23)^	*Aprendizagem baseada em equipes: um ensaio clínico randomizado na graduação em enfermagem*	Brazil	Portuguese	Randomized clinical trial	Undergraduate	Nursing students	25
E12	Kim et al. (2019)^(24)^	Effects of a patient safety course using a flipped classroom approach among undergraduate nursing students: A quasiexperimental study	South Korea	English	Quasiexperimental study	Undergraduate	Nursing students	75
E13	Beckett et al. (2017)^(25)^	A Team, Case-based Examination and Its Impact on Student Performance in a Patient Safety and Informatics Course	India	English	Cohort study	Undergraduate	Undergraduate pharmacy programs	146
E14	Thom et al. (2016)^(26)^	Advancing interprofessional patient safety education for medical, nursing, and pharmacy learners during clinical rotations	United States	English	Cohort, crosssectional study	Undergraduate	Undergraduate programs in pharmacy, medicine, and nursing	43
E15	Mariani et al. (2015)^(27)^	Improving Students’ Safety Practice Behaviors Through a Simulation-Based Learning Experience	United States	English	Nonexperimental pre-test and post-test	Undergraduate	Nursing students	175
E16	Mira et al. (2015)^(28)^	Formación en seguridad del paciente en las escuelas de medicina y enfermería en Espana	Spain	English	Observational study	Undergraduate	Nursing and medicine students	144
E17	Gordon et al. (2012)^(29)^	Non-technical skills training to enhance patient safety: a systematic review	United Kingdom	English	Systematic review	Undergraduate and graduate	Nursing faculty	22 estudos
E18	Piscotty et al. (2011)^(30)^	Integrating quality and safety competencies into undergraduate nursing using studentdesigned simulation	United States	English	Quasiexperimental study	Undergraduate	Nursing students	141
E19	Sukkari et al. (2008)^(31)^	Development and evaluation of a required patient safety course	United States	English	Pre- and postintervention study	Undergraduate	Pharmacy students	128

* *ID – Identification.*

The teaching-learning strategies related to patient safety in HEIs were presented in Chart 3.

**Chart 3:** Mapping of learning strategies on patient safety in higher education institutions, Juiz de Fora, Minas Gerais, Brazil, 2024

Learning Strategies
Clinical simulation(17,18,19,27,30)
Courses on patient safety(14,20,26,^31)^
Competency assessment^(21,22)^
Expository method^(16,23)^
Workshop^(13)^
Objective Structured Clinical Examination (OSCE)^(15)^
Flipped classroom^(24)^
Team-based case examinations^(25)^
Questionnaire application^(28)^
Role-playing^(29)^
Games on patient safety^(29)^

## DISCUSSION

This study mapped the evidence available in the literature on teaching-learning strategies related to patient safety, extensively researched in healthcare education courses. Although most of the evidence comes from nursing courses^(13,14,15,16,17,18,19,20,21,23,24,27)^, methods in medicine and pharmacy were also identified^(13,17,19,22,25,31)^. However, no publication addressed specific strategies for dental courses, highlighting a significant gap in health education. The publications presented various pedagogical methods that impact competencies, knowledge, and attitudes toward the subject.

Clinical simulation was identified as the primary teaching-learning strategy in studies E5, E6, E7, E15, and E18^(17,18,19,27,30)^. This pedagogical model was adopted in the classroom in study 18, demonstrating improvements in self-efficacy and student knowledge related to competency development^(30)^. Research from E7 and E15 noted that students appreciated working from this perspective, providing comfort in reporting or investigating incidents and adverse events during internships^(19,27)^. Studies E5 and E6 corroborated these findings and highlighted that working with designed simulations enhances learning, promoting the integration of theory and practice in undergraduate education and health services^(18,23)^.

The courses conducted during the formative process were one of the strategies identified in this scoping review. E2 and E14 stated that this alternative expands competencies and attitudes towards patient safety among nursing students^(14,26)^. It also includes a higher level of understanding of the principles and concepts for exploring teaching opportunities beyond the traditional educational model, as pointed out by studies E8 and E19^(20,31)^.

Competency-based learning assessment was shown to increase confidence among nursing students in the classroom, as evidenced by study E9^(21)^. In this instance, they observed a problem with applying theoretical aspects in a practical context and emphasized that this teaching-learning strategy could bridge such a gap in the formative process^(21)^. A study conducted with pharmacy students, E10, also emphasizes that this model is a fundamental building block for professional practice, preparing students for initial work activities^(22)^.

Regarding expository methods, a positive impact was observed in the research of E4 and E11^(16,23)^. These researchers identified little consistency in teaching approaches to patient safety in nursing programs, which are still fragmented throughout the course, lacking a specific discipline on the subject. They suggest working on this teaching-learning strategy as a team, which facilitates knowledge acquisition^(16)^.

The workshop promoted development among study participants and facilitators across all disciplines, providing students with exposure to a different context within the realm of quality and safety through the formation of interdisciplinary teams among nursing and medical students, as demonstrated in Study E1. Notable contributions include the acquisition of knowledge, attitudes, and skills related to teamwork, as well as the successful use of simulation in forming interprofessional teams^(13)^.

Knowledge, attitudes, communication, and clinical skills were assessed using the OSCE among the students. This evaluation method enhanced all these aspects, fostering the development of practical skills in patient safety. The study highlighted the importance of the OSCE as an effective tool for clinical practice, emphasizing its contribution to the training of highly competent health professionals^(15)^.

The flipped classroom and team-based exams can serve as complementary strategies. Studies E12 and E13 showed a significant increase in student competence when assessed in pre- and post-tests. Researchers associated these methods with improved student performance and enhanced self-confidence in executing skills related to medication safety^(24,25)^.

Finally, additional complementary learning strategies include the application of test questionnaires, role-playing, and games, as described in Studies E16 and E17^(28,29)^. Similar results were achieved across these studies, yet there remains a need to enhance patient safety training in both nursing and medicine, even though nursing students received more information on the topic during their undergraduate education^(28)^. The data demonstrate a favorable response from the evaluated target audience, suggesting that these pedagogical models have educational relevance^(29)^.

### Study limitations

This study highlighted a limitation worth considering: the possibility that it did not cover all available evidence sources due to restrictive inclusion criteria or the selection of specific databases for research. Therefore, the search strategy was developed by four members of the research group and a librarian, with the goal of ensuring consistent results and broadly covering the available literature to capture relevant publications.

### Contributions to the Field

As a contribution to clinical practice, this study highlights the potential to significantly add to the body of knowledge on teaching strategies related to patient safety. The results obtained can provide valuable insights for higher education institutions, enabling them to adopt various pedagogical models that demonstrate positive outcomes in student learning. This may include promoting methodologies that enhance skills, attitudes, and teamwork, as well as facilitating better integration between theoretical and practical content on patient safety.

## CONCLUSIONS

The teaching-learning strategies in patient safety at higher education institutions encompass various methodologies. Among these, clinical simulation, courses on patient safety, competency assessment, and the expository method were most frequently employed. Additionally, workshops, structured objective clinical examinations, flipped classrooms, team-based exams, test questionnaire applications, role-playing, and games have shown positive results in knowledge, attitudes, and practical skills among students and/or faculty in health-related courses. Regarding the implications of this study, the results may support the formulation of educational policies that mandate the incorporation of teaching strategies about patient safety into curricular projects, ensuring comprehensive and quality training.
