# Supplementary material for: Teaching-learning strategies on patient safety in higher education institutions: a scoping review
Source: Rev Bras Enferm. 2025 Mar 10;78(1):e20240270. doi: 10.1590/0034-7167-2024-0270 (PMC11893066; doi:10.1590/0034-7167-2024-0270)
Supplement: Translation

# Translation: Estratégias de ensino-aprendizagem sobre segurança do paciente em instituições de ensino superior: revisão de escopo

**DOI:** 10.1590/0034-7167-2024-0270pt

**Authors:** Nicole Ferreira, Vanessa Albuquerque Alvim de Paula, Marcelo Carneiro, Vilanice Alves de Araújo Püschel, Fábio da Costa Carbogim, André Luiz Silva Alvim

**Affiliations:** I Universidade Federal de Juiz de Fora. Juiz de Fora, Minas Gerais, Brasil; II Universidade de Santa Cruz do Sul. Santa Cruz do Sul, Rio Grande do Sul, Brasil; III Universidade de São Paulo. São Paulo, São Paulo, Brasil; IV Centro Brasileiro para o Cuidado à Saúde em Evidências: Centro de Excelência do JBI. São Paulo, São Paulo, Brasil

**Keywords:** Segurança do Paciente, Universidades, Educação, Aprendizagem, Revisão

## Abstract

**Objetivos:**

mapear a produção científica sobre estratégias de ensino-aprendizagem relacionadas à segurança do paciente em instituições de ensino superior nos cursos de Enfermagem, Farmácia, Medicina e Odontologia.

**Métodos:**

trata-se de uma revisão de escopo seguindo as diretrizes do *Joanna Briggs Institute* (JBI) e as recomendações do *PRISMA Extension for Scoping Reviews*. A seleção dos estudos foi realizada em bases de dados, literatura cinza e busca reversa, conduzida por dois revisores de forma independente e cegada.

**Resultados:**

foram incluídos 19 estudos, a maioria realizada no âmbito da graduação, principalmente entre discentes de Enfermagem. As estratégias de ensino-aprendizagem identificadas foram: simulação clínica, cursos, avaliação por competências e método expositivo. Além dessas, foram utilizados workshops, exame clínico objetivo estruturado, sala de aula invertida, prova em equipe, aplicação de questionários de testes, dramatização e jogos.

**Conclusões:**

as estratégias de ensino-aprendizagem mais utilizadas foram a simulação clínica e os cursos sobre segurança do paciente.

## INTRODUÇÃO

A segurança do paciente envolve a qualidade e a eficiência dos cuidados prestados aos usuários dos serviços de saúde^(1)^. Refere-se à redução, ao mínimo aceitável, de danos desnecessários que dependem, em sua maioria, do conhecimento científico e das habilidades teórico-práticas dos profissionais de saúde. Inclui a implementação de protocolos rigorosos, a promoção de uma cultura de segurança e a educação em saúde para reduzir os incidentes e eventos adversos^(2)^.

Diante disso, é fundamental que as instituições de ensino superior (IES) incluam em seus projetos pedagógicos de curso (PPC) estratégias voltadas para a formação em segurança do paciente. A capacitação do discente ao longo do seu processo formativo, seja na graduação ou pós-graduação, visa identificar e prevenir danos que possam colocar em risco a vida e a saúde dos pacientes, tais como erros de medicação, infecções relacionadas à assistência à saúde, lesões por pressão e quedas^(3)^.

Um estudo reforça que a promoção da segurança do paciente envolve uma abordagem multifacetada, a qual influencia o processo de aprendizagem durante a formação e, futuramente, garantirá a qualidade assistencial nos serviços de saúde. Quando os profissionais de saúde são incentivados por docentes durante sua trajetória acadêmica, têm maior probabilidade de fornecer o cuidado seguro e livre de danos^(4)^.

As estratégias de ensino-aprendizagem sobre segurança do paciente desenvolvidas pelas IES devem ser transversais, abrangendo todas as áreas da saúde. Essas estratégias são conceituadas como procedimentos e comportamentos escolhidos com o intuito de facilitar a aquisição, o armazenamento e a aplicação do conhecimento. Elas promovem a integração entre a teoria e a prática, incentivando a reflexão crítica e a formação contínua dos discentes e profissionais de saúde^(5)^.

Os cursos de graduação na área da saúde, tais como Enfermagem, Medicina, Farmácia, Fisioterapia, Odontologia e outras profissões que atuam em diferentes níveis de atenção à saúde, devem incluir em suas grades curriculares disciplinas específicas sobre segurança do paciente. Na pós-graduação, é importante que a oferta de capacitações e atualizações aprofunde assuntos da prática clínica para o aprimoramento das habilidades e competências do profissional^(4,6)^.

Pesquisadores alertam que ainda é preciso aprimorar o ensino sobre segurança do paciente na graduação e pós-graduação^(7,8)^. Conteúdos ministrados utilizando diferentes abordagens metodológicas poderão ser compreendidos pelos discentes como uma prioridade que impacta diretamente a saúde. No entanto, a literatura não apresenta em um único estudo quais são as estratégias de ensino-aprendizagem sobre segurança do paciente desenvolvidas e trabalhadas pelos cursos superiores. Essas pesquisas geralmente são desenvolvidas de forma unicêntrica, com amostras reduzidas, e apresentam resultados que não podem ser generalizados. Isso constitui a lacuna que justifica este estudo^(3,7,8)^.

Este estudo poderá promover reflexões acerca do impacto da educação e de suas metodologias de ensino-aprendizagem no processo formativo sobre a segurança do paciente nas IES, especialmente nos cursos de Enfermagem, Farmácia, Medicina e Odontologia, que apresentam o maior número de alunos na área

da saúde. Busca-se, portanto, identificar as estratégias de aprendizagem voltadas para o processo formativo em saúde, incluindo treinamentos, workshops e outras atividades de capacitação realizadas em sala de aula, visando o enfrentamento dos desafios relacionados à temática em diferentes cenários de atuação.

## OBJETIVOS

Mapear a produção científica sobre estratégias de ensino-aprendizagem relacionadas à segurança do paciente em IES nos cursos de Enfermagem, Farmácia, Medicina e Odontologia.

## MÉTODOS

### Aspectos éticos

Por se tratar de um estudo que incluiu apenas publicações disponíveis na literatura, não foi necessário um parecer do Comitê de Ética em Pesquisa (CEP).

### Tipo de estudo

Trata-se de uma revisão de escopo estruturada de acordo com a metodologia do JBI^(9)^ e as recomendações do *PRISMA Extension for Scoping Reviews* (PRISMA-ScR)^(10)^ para orientar a condução e redação do estudo.

### Procedimento metodológico

O objetivo, a pergunta de pesquisa, os critérios de inclusão e os métodos foram definidos e alinhados previamente em um protocolo de revisão de escopo que foi registrado e publicado na plataforma Open Science Framework (OSF), sob o número DOI: 10.17605/OSF.IO/YRWXN.

Para a elaboração da questão de pesquisa, adotou-se o mnemônico PCC, onde P = participantes, C = conceito e C = contexto. Para esta revisão, adotou-se: P = cursos da área de saúde; C = estratégias de ensino-aprendizagem sobre segurança do paciente; e C = instituições de ensino superior, tanto para cursos de graduação quanto de pós-graduação. Assim, definiu-se a seguinte questão norteadora: Quais são as estratégias de ensino-aprendizagem sobre a segurança do paciente desenvolvidas e trabalhadas nos cursos da saúde pelas IES?

Como critérios de inclusão, foram consideradas publicações abrangendo estudos analíticos e descritivos, bem como experimentais ou quase-experimentais e estudos de revisão. Os tipos de documentos considerados foram artigos científicos, monografias, dissertações e teses. Além da busca nas bases de dados, os pesquisadores conduziram uma busca reversa em todas as referências dessas pesquisas para identificar estudos adicionais. Optou-se por não impor restrições temporais ou de idioma. Foram excluídos resumos, cartas ao editor, artigos de opinião, estudos que não respondiam à questão da pesquisa, além de registros duplicados nas fontes de dados.

### Coleta e organização dos dados

A busca nas bases de dados foi realizada no dia 12 de julho de 2023 e atualizada em 2 de janeiro de 2024, seguindo uma estratégia de busca delineada com o auxílio de uma bibliotecária para localizar estudos publicados e não publicados. Palavras-chave, descritores e/ou termos livres de interesse foram utilizados para a recuperação da informação científica, considerando as particularidades de cada base de dados e a biblioteca online. Neste caso, foram incluídos artigos pagos e de acesso gratuito. A seleção de publicações na literatura cinza foi realizada no Google Acadêmico (10 primeiras páginas) e no *Open Access Theses and Dissertations* (OATD) (Quadro 1).

**Quadro 1:** Descrição das estratégias de busca para recuperação da informação científica, Juiz de Fora, Minas Gerais, Brasil, 2024

Fonte de informação	Busca realizada
PUBMED	Search: (medicine/educ*) OR (“Physical Therapists/education”[Mesh]) OR (“Education, Pharmacy”[Mesh]) OR (“Education, Dental”[Mesh]) OR (“Education, Nursing”[Mesh]) AND (course) OR (discipline) AND (patient safety/educ*)
Scopus	(TITLE-ABS-KEY (“Patient Safety” AND “Teaching” OR “Universities” not “Health professionals”) AND (course) OR (discipline) AND (patient safety/educ*)
LILACS*	*(“Patient Safety”) AND (“Teaching”) OR (“Educations”) AND (“Universities”) AND (db:(“LILACS”)*
Base de Dados em Enfermagem (BDENF)	*(“Patient Safety”) AND (“Teaching”) OR (“Educations”) AND (“Universities”) AND (db:(“BDENF”)*
Índice Bibliográfico Espanhol de Ciências da Saúde (IBECS)	*“Patient Safety” AND “Teaching” OR “Educations” AND “Universities” AND (db:(“IBECS”)*
Index Medicus do Pacífico Ocidental (WPRO)	*tw:(“Patient Safety” AND “Teaching” OR “Universities” OR “Graduation” OR “Postgraduate” not “Hospitals”) AND (db:(“WPRIM”)*
Cochrane Library	*“Patient Safety” AND “Teaching” OR “Educations” AND “Universities” NOT “Health professionals” NOT “Hospitals”*
*Scientific Electronic Library Online* (SciELO)	*“Patient Safety” AND “Teaching” OR “Universities” OR “Discipline” NOT “Health professionals” NOT “Hospitals”*
*Scholar Google*	*Patient Safety” AND “Teaching” OR “Universities” NOT “Health professionals”*
OATD	*Patient Safety AND Teaching OR Universities NOT Health professionals*

* *A estratégia de busca foi realizada utilizando os descritores nos três idiomas (inglês, português e espanhol), visto que a Literatura Latino-Americana e do Caribe em Ciências da Saúde (LILACS) é uma base de dados trilíngue.*

Em seguida, uma busca reversa foi conduzida em todas as referências e nas citações dos estudos selecionados para a extração de dados. Após realizar a estratégia de busca, as informações levantadas nas diferentes fontes foram importadas para o software Mendeley® e as duplicatas removidas. Logo em seguida, houve a importação dos registros para o software *Rayyan – Intelligent Systematic Review*, sendo utilizada para seleção e exploração dos estudos^(11)^. Esta etapa consistiu na avaliação dos títulos e resumos conforme a estratégia PCC definida nesta revisão de escopo. Posteriormente, o aprofundamento por meio de leitura minuciosa dos textos selecionados foi realizado pelos pesquisadores, que registraram os motivos de exclusão das publicações, quando necessário.

### Análise dos dados

O processo de seleção foi conduzido por dois revisores independentes, e quaisquer discordâncias foram resolvidas por um terceiro pesquisador^(9)^. As recomendações do JBI foram seguidas para a extração de dados^(12)^. As informações dos estudos selecionados e incluídos na revisão de escopo foram registradas em um formulário específico, o qual foi testado previamente nas cinco primeiras publicações identificadas durante a estratégia de busca. Não foram identificadas necessidades de ajustes ou acréscimos de informações. As variáveis consideradas foram: autor, ano de publicação, título, país do estudo, idioma, tipo de estudo, ambiente, participantes, tamanho da amostra, estratégia de aprendizagem utilizada e principais destaques.

Os resultados foram apresentados por meio de quadros e do fluxograma PRISMA, os quais responderam à pergunta de pesquisa e aos objetivos estabelecidos neste estudo. A análise dos dados foi conduzida por meio de estatística descritiva, apresentando valores absolutos e relativos em relação às publicações incluídas. Para facilitar a identificação dos estudos a serem discutidos, a letra “E” foi utilizada como identificador dos estudos.

## RESULTADOS

A Figura 1 apresenta o fluxograma PRISMA das publicações desta revisão de escopo. Identificaram-se 488 estudos nas bases de dados e na biblioteca eletrônica selecionadas para o estudo. Deste total, 19 foram incluídos após a seleção das publicações rastreadas e atenderam aos critérios de elegibilidade. Quanto à literatura cinza e à busca reversa, os estudos encontrados já estavam contidos em uma ou mais bases de dados incluídas neste estudo, não sendo caracterizados como novas pesquisas.

**Figura 1:** PRISMA dos artigos identificados e incluídos na revisão de escopo, Juiz de Fora, Minas Gerais, Brasil, 2024

O Quadro 2 mostra que a maioria dos estudos foi publicada nos anos de 2019 (31,6%) e 2020 (21,1%). O inglês predominou como idioma de publicação (94,7%), sendo os Estados Unidos o país líder em pesquisas voltadas à temática. Diversas abordagens metodológicas foram empregadas, com variações no tamanho da amostra, principalmente entre estudantes de graduação, os quais eram, na maioria, discentes de enfermagem.

**Quadro 2:** Síntese dos estudos incluídos na revisão de escopo, Juiz de Fora, Minas Gerais, Brasil, 2024 (N=19)

ID*	Autor/ano		Título	País do estudo	Idioma	Tipo de estudo	Ambiente	Participantes	Tamanho da amostra
E1	Robertson et al. (2010)^(13)^		*The Useof Simulationand aModifiedTeamSTEPPSCurriculum forMedicalandNursing Student Team Training*	Estados Unidos	Inglês	Estudo descritivo	Graduação	Discentes de enfermagem e medicina	213
E2	Lee et al. (2023)^(14)^		*Evaluatingapatient safety courseforundergraduate nursing students:Aquasi-experimental study*	Canadá	Inglês	Estudo quaseexperimental	Graduação	Discentes de enfermagem	107
E3	Yilmaz et al. (2020)^(15)^		*Theeffectiveness of scenario-based learningtodevelop patient safety behaviorin firstyear nursing students*	Canada	Inglês	Estudo quaseexperimental	Graduação	Discentes de enfermagem	351
E4	Lee et al. (2020)^(16)^		*Patient safety educationinpreregistration nursing programmesin SouthKorea*	Coreia do Sul	Inglês	Estudo descritivo	Graduação	Docentes de enfermagem	80
E5	Sanko et al. (2020)^(17)^		*Participationin aSystem-Thinking Simulation Experience ChangesAdverseEvent Reporting*	Estados Unidos	Inglês	Estudo de intervençãocontrole	Graduação	Discentes enfermagem e medicina	231
E6	Moloney et al. (2020)^(18)^		*Fourth year nursing students’ perceptions of their educational preparationinmedicationmanagement: Aninterpretativephenomenologicalstudy*	Irlanda	Inglês	Estudo qualitativo	Graduação	Discentes de enfermagem	14
E7	Packard et al. (2019)^(19)^		*ASynchronous Interprofessional Patient Safety Simulation Integrating DistanceHealthProfessions Students*	Estados Unidos	Inglês	Estudo descritivo	Graduação	Discentes de vários cursos	201
E8		Danko (2019)^(20)^	*TheEffect ofanUndergraduatePerioperativeNursing Course on Safety Knowledge*	Estados Unidos	Inglês	Estudo descritivo	Graduação	Discentes de enfermagem	44	
E9		Mbuthia et al. (2019)^(21)^	*Preregistrationnursing students’perceived confidenceinlearning about patient safety inselected Kenyan universities*	África	Inglês	Estudo transversal descritivo	Graduação	Discentes de enfermagem	194	
E10		Bajis et al. (2019)^(22)^	*Pharmacy students’ medication history taking competency: Simulationand feedbacklearning intervention*	Austrália	Inglês	Estudo de coorte	Graduação	Discentes de farmácia	144	
E11		Sakamoto et al. (2019)^(23)^	Aprendizagem baseada em equipes: um ensaio clínico randomizado na graduação em enfermagem	Brasil	Português	Ensaio clínico randomizado	Graduação	Discentes de enfermagem	25	
E12		Kim et al. (2019)^(24)^	*Effects ofapatient safety course usingaflipped classroom approach among undergraduate nursing students:Aquasi-experimental study*	Coréia do Sul	Inglês	Estudo quaseexperimental	Graduação	Discentes de enfermagem	75	
E13		Beckett et al. (2017)^(25)^	*ATeam, Case-based ExaminationandIts Impact on StudentPerformance in aPatient SafetyandInformatics Course*	Índia	Inglês	Estudo de coorte	Graduação	Cursos de graduação em farmácia	146	
E14		Thom et al. (2016)^(26)^	*Advancing interprofessional patient safety educationformedical, nursing,andpharmacy learners during clinical rotations*	Estados Unidos	Inglês	Estudo de coorte, transversal	Graduação	Cursos de graduação em farmácia, medicina e enfermagem	43	
E15		Mariani et al. (2015)^(27)^	*Improving Students’ Safety Practice Behaviors ThroughaSimulation-Based Learning Experience*	Estados Unidos	Inglês	Pré-teste e pós-teste não experimental	Graduação	Discentes de enfermagem	175	
E16		Mira et al. (2015)^(28)^	*Formaciónenseguridad del pacienteenlas escuelas de medicina y enfermeríaenEspana*	Espanha	Inglês	Estudo observacional	Graduação	Discentes enfermagem e medicina	144	
E17		Gordon et al. (2012)^(29)^	*Non-technical skills trainingtoenhance patient safety:asystematic review*	Reino Unido	Inglês	Revisão sistemática	Graduação e pósgraduação	Docentes de enfermagem	22 estudos	
E18		Piscotty et al. (2011)^(30)^	*Integrating qualityandsafety competencies into undergraduate nursing using student-designed simulation*	Estados Unidos	Inglês	Estudo quaseexperimental	Graduação	Discentes de enfermagem	141	
E19		Sukkari et al. (2008)^(31)^	*Developmentandevaluation ofarequired patient safety course*	Estados Unidos	Inglês	Estudo pré e pós-intervenção	Graduação	Discentes de farmácia	128	

* *ID – Identificação.*

As estratégias de ensino-aprendizagem sobre segurança do paciente em IES foram apresentadas no Quadro 3.

**Quadro 3:** Mapeamento das estratégias de aprendizagem sobre segurança do paciente em instituições de ensino superior, Juiz de Fora, Minas Gerais, Brasil, 2024

Estratégias de aprendizagem	
Simulação clínica(17,18,19,27,30)	
Cursos sobre segurança do paciente^(14,20,26,31)^	
Avaliação por competências^(21,22)^	
Método expositivo^(16,23)^	
*Workshop* ^(13)^
Exame Clínico Objetivo Estruturado (OSCE)^(15)^
Sala de aula invertida^(24)^
Prova em equipe, baseada em casos^(25)^
Aplicação de questionário de testes^(28)^
Dramatização^(29)^
Jogos sobre segurança do paciente^(29)^

## DISCUSSÃO

Este estudo mapeou as evidências disponíveis na literatura sobre as estratégias de ensino-aprendizagem em segurança do paciente, amplamente investigadas em cursos da área da saúde. Embora a maioria das evidências seja proveniente do curso de enfermagem^(13,14,15,16,17,18,19,20,21,23,24,27)^, abordagens na medicina e farmácia também foram identificadas^(13,17,19,22,25,31)^. Contudo, nenhuma publicação abordou estratégias específicas para cursos de odontologia, evidenciando uma lacuna importante para a área do ensino na saúde. As publicações apresentaram diferentes métodos pedagógicos, que impactam nas competências, no conhecimento e nas atitudes em relação ao tema.

A simulação clínica foi identificada como a principal estratégia de ensino-aprendizagem nos estudos de E5, E6, E7, E15 e E18^(17,18,19,27,30)^. Esse modelo pedagógico foi adotado em sala de aula no estudo 18, evidenciando melhorias na autoeficácia e no conhecimento dos alunos relacionados ao desenvolvimento das competências^(30)^. As pesquisas de E7 e E15 afirmam que os discentes apreciaram trabalhar nessa perspectiva, proporcionando conforto para relatar ou investigar incidentes e eventos adversos na prática de estágio^(19,27)^. Os estudos de E5 e E6 corroboraram esses achados e destacaram que trabalhar com simulação projetada favorece o aprendizado, promovendo a integração da teoria e da prática na graduação e no serviço de saúde^(18,23)^.

Os cursos ministrados durante o processo formativo foram uma das estratégias identificadas nesta revisão de escopo. E2 e E14 afirmam que essa alternativa amplia as competências e atitudes em relação à segurança do paciente entre estudantes de enfermagem^(14,26)^. Inclui-se, também, um maior nível de compreensão acerca dos princípios e conceitos para explorar oportunidades de ensino além do modelo educacional tradicional, conforme apontado pelos estudos de E8 e E19^(20,31)^.

A avaliação de aprendizagem por competências demonstrou aumentar a confiança dos estudantes de enfermagem em sala de aula, conforme evidenciado no estudo de E9^(21)^. Neste caso, eles observaram um problema com a aplicação dos aspectos teóricos no contexto prático e reforçaram que essa estratégia de ensino-aprendizagem pode diminuir tal lacuna no processo formativo^(21)^. Uma pesquisa realizada com discentes do curso de farmácia, E10, também ressalta que esse modelo constitui uma construção em blocos fundamental para a prática profissional, preparando o aluno para a atividade de trabalho inicial^(22)^.

Em relação aos métodos expositivos, observou-se um impacto positivo nas pesquisas de E4 e E11^(16,23)^. Esses pesquisadores identificaram pouca consistência nas abordagens de ensino sobre segurança do paciente em programas de enfermagem, as quais ainda estão fragmentadas ao longo do curso, sem uma disciplina específica sobre o tema. Eles sugerem trabalhar essa estratégia de ensino-aprendizagem em equipe, o que favorece a apreensão do conhecimento^(16)^.

O workshop promoveu o desenvolvimento entre os participantes do estudo e os facilitadores em todas as disciplinas, proporcionando aos discentes exposições a um contexto diferente no âmbito da qualidade e segurança, através da formação de equipes interdisciplinares entre discentes de enfermagem e medicina, como demonstrado no estudo E1. Dentre as contribuições, destacam-se a aquisição de conhecimentos, atitudes e habilidades relacionadas ao trabalho em equipe, bem como o uso bem-sucedido de simulação na formação de equipe interprofissional^(13)^.

O conhecimento, as atitudes, a comunicação e as habilidades clínicas foram verificados por meio da utilização do OSCE entre os discentes. Todos os aspectos foram aprimorados por esse método de avaliação, proporcionando o desenvolvimento de aptidões práticas em segurança do paciente. Esse estudo ressaltou a importância do OSCE como uma ferramenta eficaz para a prática clínica, destacando sua contribuição para a formação de profissionais de saúde altamente competentes^(15)^.

Já a sala de aula invertida e a prova em equipe podem ser utilizadas como estratégias complementares. Os estudos de E12 e E13 demonstraram um aumento significativo na competência dos estudantes quando avaliados em pré e pós-testes. Esses métodos foram associados, pelos pesquisadores, ao melhor desempenho dos alunos e à melhoria da autoconfiança na execução de habilidades relacionadas à segurança medicamentosa^(24,25)^.

Por fim, outras estratégias de aprendizagem complementares incluem a aplicação de questionários de testes, dramatização e jogos, conforme os estudos de E16 e E17^(28,29)^. Resultados similares foram alcançados entre as pesquisas, mas ainda se percebe a necessidade de melhorar a formação em segurança do paciente tanto na enfermagem quanto na medicina, embora os discentes de enfermagem tenham recebido mais informações sobre o tema na graduação^(28)^. Os dados evidenciam uma resposta favorável do público-alvo avaliado, sugerindo que esses modelos pedagógicos têm relevância educacional^(29)^.

### Limitações do estudo

Este estudo destacou uma limitação que merece consideração: a possibilidade de não abranger todas as fontes de evidência disponíveis, devido a critérios de inclusão restritos ou à seleção de bases de dados específicas para pesquisa. Portanto, a estratégia de busca foi desenvolvida por quatro profissionais pertencentes a um grupo de pesquisa e uma bibliotecária, com o objetivo de garantir resultados consistentes e abranger amplamente a literatura disponível para capturar as publicações relevantes.

### Contribuições para a Área

Como contribuição para a prática clínica, destaca-se o potencial de agregar significativamente ao corpo de conhecimento sobre as estra -tégias de ensino relacionadas à segurança do paciente. Os resultados obtidos podem fornecer subsídios valiosos para as IES, permitindo que estas adotem diversos modelos pedagógicos que demonstrem resultados positivos na aprendizagem dos alunos. Isso pode incluir a promoção de metodologias que aprimorem habilidades, atitudes e trabalho em grupo, além de facilitar uma melhor integração entre conteúdos teóricos e práticos sobre a segurança do paciente.

## CONCLUSÕES

As estratégias de ensino-aprendizagem em segurança do paciente nas IES incluem diferentes metodologias. Dentre elas, a simulação clínica, os cursos sobre segurança do paciente, a avaliação por competências e o método expositivo foram os mais frequentes. Além dessas, workshops, exame clínico objetivo estruturado, sala de aula invertida, prova em equipe, aplicação de questionário de testes, dramatização e jogos demonstraram resultados positivos no conhecimento, na atitude e na habilidade teórico-práticas entre discentes e/ou docentes dos cursos da área de saúde. Em relação aos desdobramentos deste estudo, os resultados poderão subsidiar a formulação de políticas educacionais que promovam a incorporação obrigatória de estratégias de ensino sobre segurança do paciente nos PPC, garantindo uma formação abrangente e de qualidade.
